# Self-adherence to post-colonoscopy consults in patients undergoing diagnostic colonoscopy: Findings from a cross-sectional, quantitative survey at a South African quaternary hospital

**DOI:** 10.1371/journal.pone.0288752

**Published:** 2023-07-18

**Authors:** Yoshan Moodley, Jacqueline van Wyk, Yuming Ning, Steven Wexner, Cathrine Gounden, Vasudevan Naidoo, Shakeel Kader, Alfred I. Neugut, Ravi P. Kiran

**Affiliations:** 1 Gastrointestinal Cancer Research Group, Nelson R. Mandela School of Medicine, University of KwaZulu-Natal, Durban, South Africa; 2 Faculty of Medicine and Health Sciences, Stellenbosch University, Cape Town, South Africa; 3 Department of Health Sciences Education, University of Cape Town, Cape Town, South Africa; 4 School of Clinical Medicine, Nelson R. Mandela School of Medicine, University of KwaZulu-Natal, Durban, South Africa; 5 Department of Surgery, Columbia University, New York, NY, United States of America; 6 Ellen Leifer Shulman and Steven Shulman Digestive Disease Center, Cleveland Clinic Florida, Weston, Florida, United States of America; 7 Department of Medicine and Herbert Irving Comprehensive Cancer Center, Columbia University, New York, NY, United States of America; University of Botswana School of Medicine, BOTSWANA

## Abstract

Post-colonoscopy consults empower patients to make informed decisions around their subsequent treatment, and non-compliance with these consults (“no-shows”) hinders disease management. There is a paucity in the literature regarding self-adherence to post-colonoscopy consults in resource-limited settings such as South Africa. An understanding of self-adherence to post-colonoscopy consults in this setting is required to establish whether improved interventions are needed, and what specific elements of self-adherence should be addressed with these interventions. The objective of this hypothesis-generating, cross-sectional, quantitative survey was to conduct a baseline assessment of cognitive, motivational, social, and behavioural variables related to self-adherence to post-colonoscopy consults in patients who underwent diagnostic colonoscopy at a South African quaternary hospital. The Adherence Determinants Questionnaire (ADQ) was administered in 47 patients to establish a baseline assessment of elements related to self-adherence to post-colonoscopy consults, including interpersonal aspects of care, perceived utility, severity, susceptibility, subjective norms, intentions, and supports/barriers. ADQ scores were transformed to a percentage of the maximum score for each element (100.0%). The overall mean transformed ADQ score was 57.8%. The mean transformed scores for specific ADQ components were as follows: subjective norms (40.8%), perceived severity (55.4%), perceived utility (56.6%), intentions (59.4%), supports/barriers (59.9%), interpersonal aspects (62.2%), and perceived susceptibility (65.9%). There were no statistically significant differences in overall mean transformed ADQ scores and individual ADQ elements across categories of participant age (p-values ranging between 0.180 and 0.949 when compared between participants ≤40 years and >40 years old), gender (p-values ranging between 0.071 and 0.946 when compared between males and females), and race (p-values ranging between 0.119 and 0.774 when compared between Black Africans and non-Black Africans). Our findings suggest a general need for appropriate interventions to improve self-adherence to post-colonoscopy consults in our setting.

## Introduction

Diagnostic colonoscopy is the method of choice for investigating lower gastrointestinal disease pathologies [1, 2]. A finalised report from a diagnostic colonoscopy, including the histopathology findings from any biopsies performed, will not be available on the day of the procedure. Thus, it is normal practice for the attending physician to arrange a consult with a patient several days or perhaps even several weeks following a diagnostic colonoscopy [3]. During this outpatient clinic engagement (hereafter referred to as the “Post-colonoscopy consult”), the physician may share information on the findings of the diagnostic colonoscopy and discuss subsequent treatment options with a patient [4]. This information can be useful in improving the patient’s decision-making around the treatment of his/her gastrointestinal condition and future follow-up [5]. The post-colonoscopy consult is even more crucial when the diagnostic findings are suggestive of conditions which necessitate timely linkage to treatment [6, 7]. This is particularly relevant in the context of colorectal cancer, which is associated with high levels of morbidity and mortality [8]. Although studies which report specifically on post-colonoscopy consult no-shows are rare, clinic no-show rates from general gastroenterology outpatient settings in the United Kingdom range from 11.3% to 14% [9, 10]. Researchers from the United States reported slightly lower no-show rates at 8.4% [11]. Therefore, non-attendance at a scheduled post-colonoscopy consult (also referred to as “no-shows”) can have important implications for the effective clinical management of lower gastrointestinal disease.

A systematic review of studies on compliance with colonoscopy by McLachlan and colleagues (mostly comprised of studies conducted in high-income countries such as the USA, UK, Canada, and Australia) reported that patients’ anxiety, fear of pain/discomfort from the procedure, concerns about a long recovery time following the procedure, perceived accuracy of a colonoscopy finding, lack of knowledge/awareness of colorectal cancer, and the perceived benefits of undergoing a colonoscopy as the most important personal barriers/facilitators to colonoscopy uptake [12]. The same authors also reported that logistical challenges (transport and costs associated with attending an outpatient clinic for a colonoscopy), competing health concerns, the doctor-patient relationship (including adequate communication between the doctor and the patient) were the most important practical and health system barriers/facilitators to colonoscopy uptake [12]. It is likely that several of the barriers and facilitators of compliance with colonoscopy procedure itself might also play a role in compliance with the post-colonoscopy consult.

There are no published research studies on self-adherence to post-colonoscopy consults in South Africa, a country which is currently facing an increasing burden of gastrointestinal conditions such as colorectal cancer. This cancer is now ranked amongst the most important cancers in South Africa, both in terms of disease incidence and cancer-related mortality [13, 14]. An understanding of self-adherence to post-colonoscopy consults in this setting is required to establish whether improved interventions are required to increase self-adherence, and what specific elements of self-adherence would need to be addressed with these interventions. The objective of this hypothesis-generating, cross-sectional, quantitative survey was to conduct a baseline assessment of cognitive, motivational, social, and behavioural variables related to self-adherence to post-colonoscopy consults in patients who underwent diagnostic colonoscopy at a South African quaternary hospital.

## Methods

### Study design and setting

This cross-sectional, quantitative survey included 47 consecutive patients (hereafter referred to as “the study sample”) who had a diagnostic colonoscopy performed at the Inkosi Albert Luthuli Central Hospital (IALCH) in Durban, South Africa between 1 March 2022 and 30 April 2022. Rather than a being hypothesis-testing study, this study was meant to be a hypothesis-generating study and provide a preliminary indication of self-adherence to post-colonoscopy consults to inform a larger hypothesis-testing study in the near future. IALCH is a government-funded healthcare facility and provides specialist or quaternary-level medical and surgical services to the population of the KwaZulu-Natal Province on the east coast of South Africa. Patients attending IALCH are usually referred from lower-level regional healthcare facilities. The population of the KwaZulu-Natal Province is diverse and primarily comprised of persons classified as Black African (87%), Asian (persons with ancestry from the Indian subcontinent, 7%), and Caucasian (4%). The waiting period between attending the booking clinic at IALCH and the diagnostic colonoscopy procedure is usually 4 weeks. A diagnostic colonoscopy usually takes between 20 and 30 minutes to complete. The colonoscopy report is generated on the same day as the procedure. The biopsy results are received from the pathology laboratory between 2 and 4 weeks after the colonoscopy was performed, and a post-colonoscopy consult is arranged as soon as possible thereafter for the patient. Currently, there are no reminders or notifications sent to patients regarding scheduled colonoscopy appointments and post-colonoscopy consults at IALCH, as there are insufficient resources to support this.

### Survey participants

Colonoscopies are performed as outpatient procedures at IALCH. The survey participants were patients identified from the gastroenterology outpatient clinic waiting room at IALCH on the day of their colonoscopy. All patients were surveyed prior to their colonoscopy. All the patients attending the clinic on the days that the survey was conducted were there to undergo a diagnostic colonoscopy. The eligibility criteria for this study were as follows: patients must have been aged older than 18 years at the time that they were surveyed, patients were willing and able to provide written informed consent to participate in the survey, and completed all survey questions. Correctional services patients, non-South African citizens, and patients who did not speak English or the local isiZulu Languages were excluded.

### Survey instrument

The Adherence Determinants Questionnaire (ADQ), a survey instrument developed by DiMatteo and colleagues to measure patient self-adherence to cancer prevention interventions and cancer treatments, was administered to all study participants [15]. The choice of survey instrument was well suited to our study since colonoscopy is an important tool used for diagnosing colorectal cancer in our setting [1]. The ADQ consists of seven elements, which were identified by DiMatteo et al., as being related to patient adherence to recommendations for both the prevention and treatment of cancer: Interpersonal aspects of medical care received, beliefs around the severity of cancer, beliefs around the perceived utility of the recommended treatments, subjective norms regarding adherence, intentions to adhere to the recommended treatments, and the presence of supports and absence of barriers to adherence [15]. Interpersonal aspects of care are assessed through eight questions, all of which are related to the physician-patient relationship and/or communication between the physician and the patient. Perceived utility is assessed through eight questions related to the benefits, costs, and perceived efficacy of the recommended treatment. Perceived susceptibility is assessed through four questions on the participant’s perceived risk of cancer in the future. Subjective norms are assessed through six questions around relative’s and friend’s thoughts on the recommended treatment plan. Intentions are assessed through four questions on the participant’s personal intention to comply with the recommended treatment. Lastly, supports and barriers are assessed through four questions around challenges and facilitators to the participant complying with his/her recommended treatment plan. The specific questions for each ADQ element are detailed in the original manuscript by DiMatteo and colleagues [15]. Each of the seven elements included in the ADQ has a point allocation based on a Likert Scale rating: Interpersonal aspects of care (8 to 40 points), perceived utility (8 to 40 points), severity (4 to 20 points), susceptibility (4 to 20 points), subjective norms (-18 to 18 points), intentions (4 to 20 points), and supports or barriers (4 to 20 points). The total ADQ score can range between 14 and 178 points [15]. Survey responses were transformed into a percentage of the maximum obtainable score for each element (Transformed ADQ score, maximum = 100.0%). The ADQ demonstrates appreciable reliability (Cronbach’s alpha = 0.65–0.85) [15].

### Data analysis

The study data was analysed with descriptive statistics and t-tests. Results of the descriptive statistical analysis are presented as frequencies and percentages or means with standard deviation (SD). The results of the t-test analysis, in which mean transformed ADQ scores were compared across categories of participant age, gender, and race are presented as means with SD and a p-value. Statistical significance was set at p<0.05. The descriptive and comparative statistical analyses were performed with R version 4.1.1 (R Foundation for Statistical Computing, Vienna, Austria). The de-identified dataset used in this analysis is provided as a supplemental file, S1 Dataset.

### Study ethical approval

Ethical approval for this research was granted by the University of KwaZulu-Natal, South Africa (Protocol BREC/00002520/2021).

## Results

A description of the study sample is provided in Table 1. The mean age of the study sample was 48.7 (SD: 17.8) years old. A total of 31 participants (66.0%) were aged >40 years old. Males accounted for just under half of the study sample (21 participants, 44.7%). The study sample was comprised of 26 Black African participants (55.3%), 20 Asian participants (42.6%), and 1 Caucasian participant (2.1%). Race was subsequently categorised as Black African or non-Black African (Asian or Caucasian) to facilitate a comparative statistical analysis of transformed ADQ scores by race group. Based on this classification, there were 26 Black African (55.3%) and 21 non-Black African (44.7%) participants in the study sample.

**Table 1:** Description of the study sample (N = 47).

Characteristic	Summary statistic
**Age**	
Mean in years (SD)	48.7 (17.8)
Age >40 years old, n (%)	31 (66.0)
**Gender**	
Male, n (%)	21 (44.7)
Female, n (%)	26 (55.3)
**Race**	
Black African, n (%)	26 (55.3)
Asian, n (%)	20 (42.6)
Caucasian, n (%)	1 (2.1)

**Fig 1:**
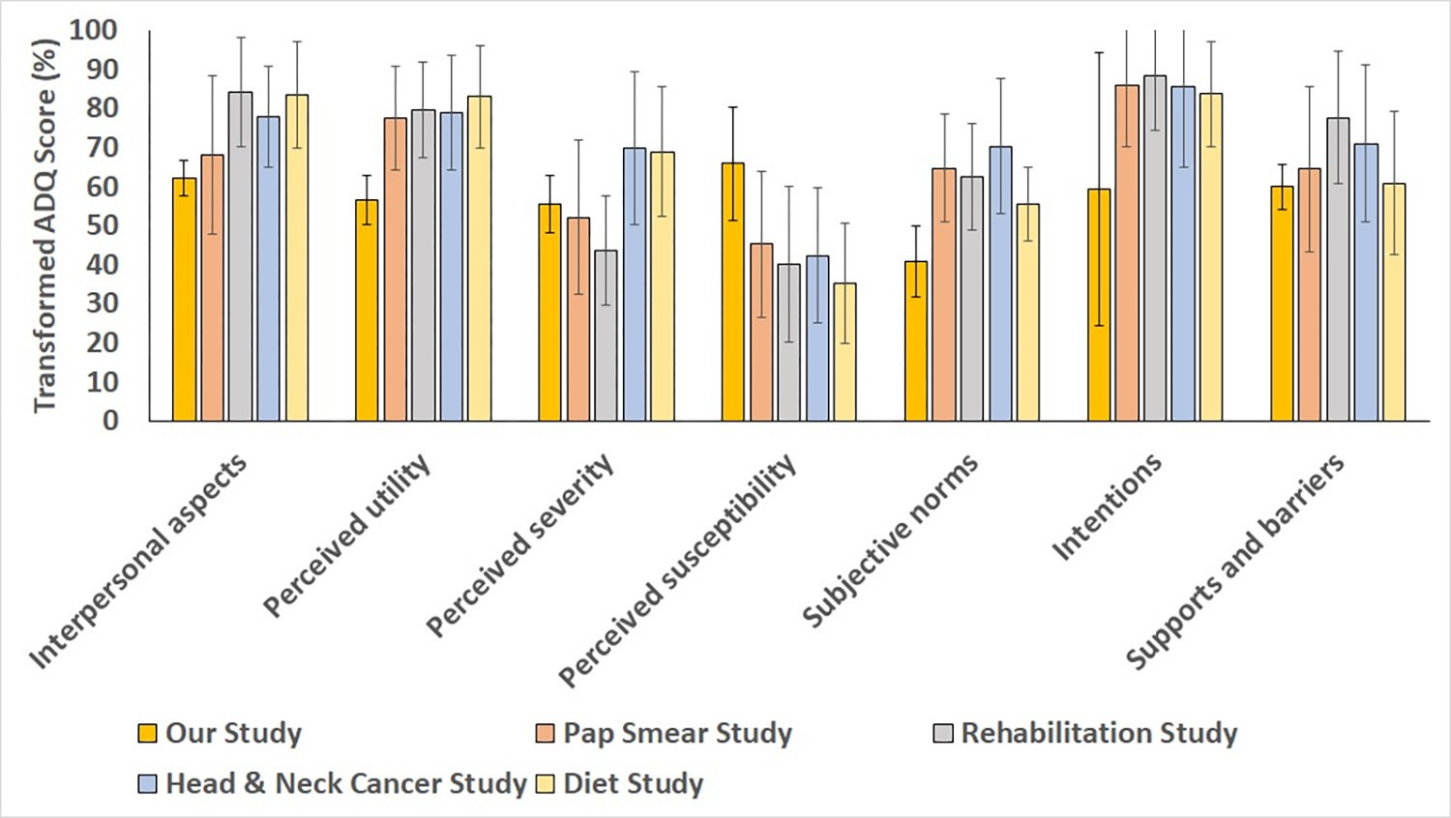
Mean transformed ADQ scores, % (SD) for each ADQ component in our study sample and the validation studies reported by DiMatteo and colleagues.

A summary of mean transformed ADQ scores (% of the maximum theoretical score for each score element, 100.0%) across the entire study sample is provided in Fig 1. The overall mean transformed ADQ score for the study sample was 57.8 (SD: 4.5). The overall mean transformed scores for individual components of the ADQ ranged from 40.8 (Subjective norms) to 65.9 (Perceived susceptibility). Fig 1 also presents the findings from validation studies of the ADQ (Mean transformed ADQ scores and SD) that were reported in the original ADQ manuscript by DiMatteo and colleagues [15]. This included studies on outpatient clinic follow-ups for abnormal Pap smears, a rehabilitation intervention in cancer patients, smoking cessation in patients with head/neck cancer, and behavioural modification involving a low-fat, high-fiber diet [15]. With a few exceptions, most of the ADQ scores in our study were similar to the ADQ scores reported in the validation studies conducted by DiMatteo and colleagues (based on overlapping SDs).

**Fig 2:**
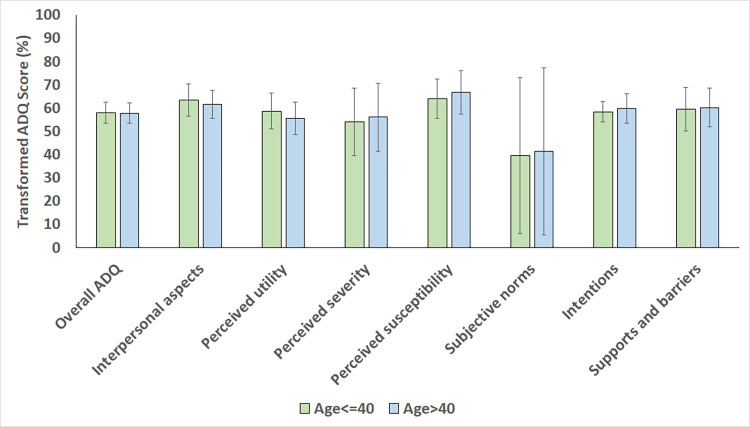
Mean transformed ADQ scores, % (SD) for each ADQ component in the study sample, compared across category of age (Age ≤40 years vs. Age >40 years).

**Fig 3:**
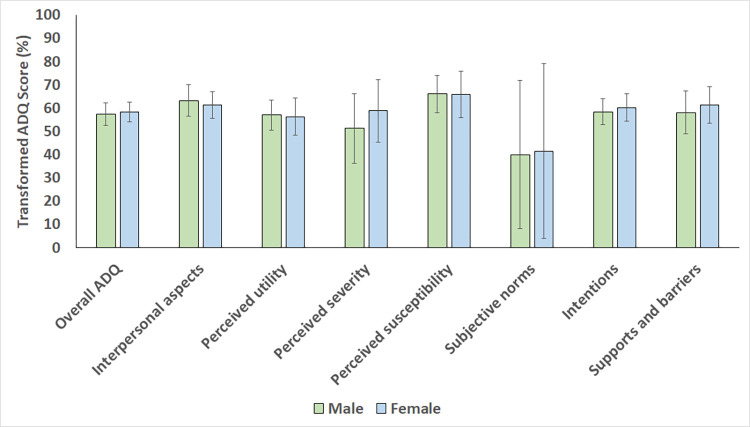
Mean transformed ADQ scores, % (SD) for each ADQ component in the study sample, compared across category of gender.

**Fig 4:**
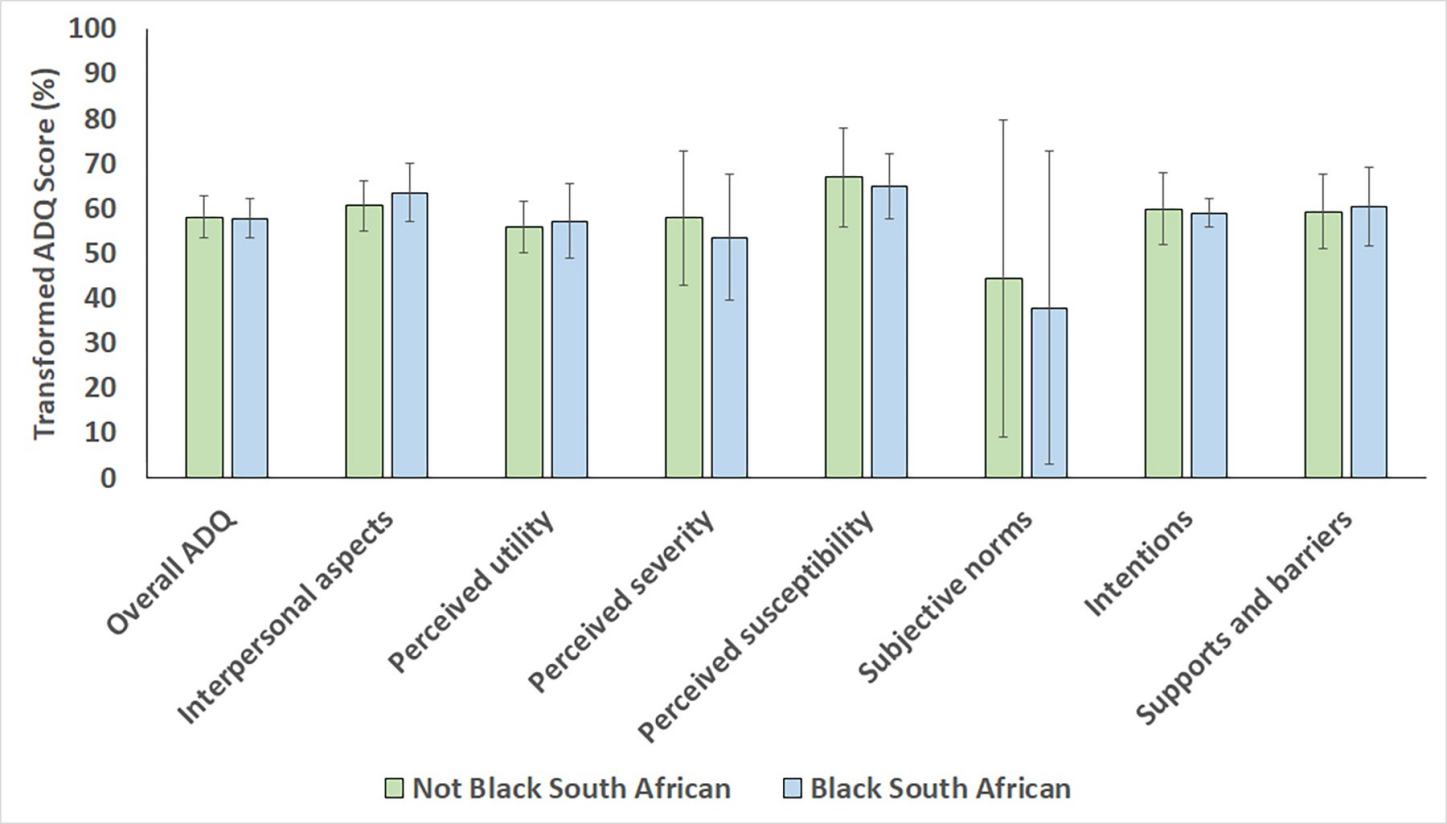
Mean transformed ADQ scores, % (SD) for each ADQ component in the study sample, compared across category of race group.

The findings from the comparative statistical analysis are presented in Figs 2–4. There were no statistically significant differences in overall mean transformed ADQ scores and individual ADQ elements across categories of participant age (p-values ranging between 0.180 and 0.949 when compared between participants ≤40 years and >40 years old), gender (p-values ranging between 0.071 and 0.946 when compared between males and females), and race (p-values ranging between 0.119 and 0.774 when compared between Black Africans and non-Black Africans).

## Discussion

The findings of this study suggest that there is a general need for appropriate interventions to improve self-adherence to post-colonoscopy consults in our setting. The ADQ provides an indication of self-adherence to medical treatment by measuring interpersonal aspects of care, perceived utility, perceived severity, perceived susceptibility, subjective norms, intentions, and supports or barriers [15]. Thus, improvements in the majority or all of these elements would contribute towards an overall improvement in self-adherence to post-colonoscopy consults in patients undergoing diagnostic colonoscopy. Improvements to interpersonal aspects of care could be achieved by encouraging better communication and information sharing among physicians/clinicians at gastroenterology clinics/clinicians at the referral hospitals/general practitioners and their patients [16]. Indeed, the published literature does confirm that positive health outcomes are fostered by improved physician-patient communication, through patient-directed or physician-directed approaches [17, 18]. In its simplest form, a patient-directed approach could involve optimising patient participation during the initial pre-colonoscopy consult, by providing an opportunity for patients to ask questions around their gastrointestinal ailment and sharing their expectations around their post-colonoscopy consults and subsequent treatment plan [16]. A physician-directed approach could involve a skills training programme, which would impart the most effective ways for a physician build a rapport with a patient and communicate the importance of adhering to post-colonoscopy consults [16]. An example of a communication skills training programme is “Oncotalk”, a four-day workshop for US postgraduate medical trainees covering content on developing a relationship with the patient, dealing with uncertainty, giving bad news, discussing transition to palliative care, and discussing do-not-resuscitate orders [19]. The programme was extremely successful and significantly improved physician-patient communication [19]. It is likely that a similar communication skills training programme for gastroenterologists would also yield positive results.

Perceived utility (benefits/costs and efficacy), perceived severity, and perceived susceptibility are core concepts of the Health Beliefs Model, which is the theoretical framework that is most widely used to explain an individual’s readiness to act on a health-related problem [20]. Knowledge and awareness of a particular disease condition is one of the main cues to action that underpin the Health Beliefs Model—if individuals do not demonstrate an acceptable level of understanding around a disease condition, then they are unable to fully recognize the potential threat and are unlikely to take any action to address the disease condition [20]. Therefore, the ADQ element “intentions” is also linked to perceived utility, perceived severity, and perceived susceptibility; and improving knowledge and awareness around a particular disease condition is likely to be beneficial in addressing these four elements of self-adherence to treatment. Large-scale public health campaigns targeting gastrointestinal diseases are currently lacking in South Africa. Thus, an urgent need exists in this setting for appropriate educational material to raise levels of knowledge around serious gastrointestinal diseases amongst at-risk populations which also emphasizes the importance of adhering to a treatment management plan, including post-colonoscopy consults. The financial costs of post-colonoscopy consult attendance to the patient must also be acknowledged. IALCH is a government-funded facility, and like most other government-funded facilities in South Africa, serves a large proportion of the population who cannot afford to access private healthcare services. While accessing medical/surgical care at government-funded facilities is free for all South African patients, there are still some out-of-pocket expenses which patients might be liable for. There are only a few facilities offering medical/surgical services at the tertiary- and quaternary-level, and most of these are in metropolitan areas [21]. Thus, some patients might have to incur travel costs to receive medical/surgical care, and these travel costs are higher the further a patient resides from the healthcare facility [22, 23]. Furthermore, travel to the hospital and the clerking process when patient arrives at the hospital might take several hours to complete. This might cause some patients to incur a loss of income on the day that they are required to attend a post-colonoscopy consult, because they might have to take time off from work to attend the consult. To defray some of the financial costs to the patient, consideration must be given to decentralizing the post-colonoscopy consult to lower-level facilities closer to the patient’s place of residence [24, 25], adopting telemedicine methods in gastroenterology units [26], and arranging a dedicated hospital bus service to facilitate attendance of a scheduled outpatient consult [27].

The influence of close family or friends is a key factor related to self-adherence to treatment and is assessed under the ADQ element “subjective norms” [15]. It is the cultural norm in some African settings for close family members to be involved in the decision-making processes around an individual’s treatment [28]. It is important that this be taken into consideration when patients present for their colonoscopies, and a close family member should be encouraged to accompany patients to the gastroenterology clinic on the day of the procedure. This can be used as an opportunity by the physician to raise levels of knowledge around gastrointestinal diseases amongst close family members of a patient and clarify misconceptions around disease pathologies and treatment options. Close family and friends also have a role in providing support and motivating patients with more serious gastrointestinal diseases, such as cancer, to continue with their recommended treatment [29, 30]. In the absence of close family and friends, it is advisable that options for support include individuals from peer groups who have been successfully treated [31, 32].

This research was not without limitations. The study sample size was small and did not facilitate a more complex statistical analysis. Given that we were conducting our study in a resource-constrained setting, we also expected a higher non-compliance rate in our patient population when planning this study. However, we found that our non-compliance rate was similar to that reported for gastroenterology clinic outpatient visits in high-income countries (we observed a 10.3% non-compliance rate in our study). This prevented us from conducting a comparison of ADQ scores between patients who complied with their recommended consult and those who did not, as our study sample size was too small to accomplish this. It is possible that since IALCH is a higher-level facility, patients in our study might have been more interested about their health and were more motivated to attend their scheduled consult than what may be the case for patients who attend lower-level healthcare facilities. Therefore, self-adherence to post-colonoscopy consults might be different in patients attending lower-level healthcare facilities. This is a limitation which might impact the external validity of our findings and is something that must be taken into account when conducting further research on this topic. This study only involved participants from the South African public healthcare sector, which serves an estimated 85% of the South African population [33]. As such, measures of self-adherence to post-procedural consults amongst the remaining 15% of the South African population who access private healthcare services could not be established as part of this research, and must also be taken into account in future studies to improve the external validity of the findings. Regrettably, due to resource constraints for this study, the participants were not identified and prepared for the survey before the day of their procedure. This might have influenced some of the participants’ responses to the survey questions. Lastly, given the small number of defaulters in our study, the reasons for defaulting on scheduled post-procedural consults were not investigated, as it would be inappropriate to draw general conclusions around reasons for non-compliance with a scheduled consult based only on the responses from a small group of defaulters.

In conclusion, improvement in all elements of self-adherence to post-colonoscopy consults should be encouraged in this setting, irrespective of patient age, gender, or race. More research is required to identify the most appropriate interventions to improve self-adherence to post-colonoscopy consults in resource-constrained South Africa. These studies should involve larger sample sizes, include participants attending private healthcare facilities, and have resources in place to follow-up on patients to establish no-show rates and reasons for non-compliance with scheduled post-colonoscopy consults.

## Supporting information

S1 Dataset De-identified dataset used in this analysis. (XLSX)
